# Changes in the urinary proteome of rats after short-term intake of magnesium L-threonate(MgT)

**DOI:** 10.3389/fnut.2023.1305738

**Published:** 2023-12-21

**Authors:** Ziyun Shen, Minhui Yang, Haitong Wang, Yuqing Liu, Youhe Gao

**Affiliations:** Beijing Key Laboratory of Gene Engineering Drug and Biotechnology, College of Life Sciences, Beijing Normal University, Beijing, China

**Keywords:** urine, proteomic, magnesium, nutrients, mineral elements

## Abstract

**Introduction:**

Magnesium (Mg) is an important mineral in living organisms. Magnesium has multiple functions in the human body, wherein it plays an important therapeutic and preventive role in a variety of diseases.

**Methods:**

Urine samples of rats before and after gavage of magnesium L-threonate (MgT) were collected, and the urinary proteome was identified using the LC-MS/MS technique and analyzed using various databases.

**Results and discussion:**

The results illustrated that the urinary proteome of rats was significantly altered after short-term intake of magnesium supplements and that the differential proteins and the biological functions were related to magnesium. This study innovatively establishes a method to study nutrients from the perspective of urine proteomics. This work demonstrates that the urinary proteome is capable of reflecting the effects of nutrient intake on the organism in a more systematic and comprehensive manner and has the potential to provide clues for clinical nutrition research and practice.

## 1 Introduction

Magnesium (Mg) is an essential mineral for living organisms. Magnesium is found in the body mainly in the form of Mg^2+^. In 1980, Theodor Günther proposed that magnesium acts as a cofactor in more than 300 enzymatic reactions in the body (1). Currently, the Enzyme Database lists over 600 enzymes in which magnesium act as cofactors and an additional 200 enzymes in which magnesium act as activators (2–4). Magnesium is essential for adenosine triphosphate (ATP) metabolism, DNA and RNA synthesis, and protein synthesis (5). In addition, magnesium plays a key role in the regulation of many physiological functions, including muscle contraction, blood pressure regulation, insulin metabolism, cardiac excitability, vasodilatory tone, neurotransmission and neuromuscular transmission (6).

Based on its many functions in the body, magnesium plays an important role in the prevention and treatment of many diseases. Low magnesium levels have been linked to many chronic and inflammatory diseases, such as Alzheimer’s disease, asthma, attention deficit hyperactivity disorder (ADHD), insulin resistance, type 2 diabetes, hypertension, cardiovascular disease, migraines, and osteoporosis (7).

Magnesium is the fourth most abundant mineral in the human body. Assessing magnesium status in the body is difficult because most magnesium is located intracellularly or in bone. The most common test used in clinical medicine to rapidly assess changes in magnesium status is the serum magnesium concentration, but there is little correlation between serum magnesium levels and systemic magnesium levels or concentrations in specific tissues (8, 9).

Humans need a regular intake of magnesium to prevent magnesium deficiency or hypomagnesemia. Many studies have demonstrated the beneficial effects of magnesium supplementation. However, since the recommended daily intake (RDI) of magnesium varies, it is difficult to define the exact optimal intake. According to the Dietary Guidelines for Chinese Residents, the recommended nutrient intake (RNI) for magnesium is 330 mg per day, and the tolerable upper intake level (UL) is 700 mg/d (10). According to the U.S. Food and Nutrition Board, the recommended daily intake of magnesium for adult males and females is 420 mg and 320 mg, respectively (4).

Approximately 10% of daily magnesium intake comes from drinking water (11); green vegetables, nuts, seeds, and unprocessed grains are rich sources of magnesium; in addition, some magnesium is found in fruits, fish, meat, and dairy products (5). Due to the consumption of processed foods and the consumption of softened water, the magnesium intake of the population in most countries is below the recommended amount (4, 12).

There are many types of magnesium supplements that can add extra magnesium to the diet. Magnesium L-threonate(MgT), so called Bis[(2R,3S)-2,3, 4-trihydroxybutanoato-alphao,Alphao]Magnesium, is a magnesium compound that has been found in recent years. The molecular formula is C8H14MgO10.

In 2010, Slutsky et al. (13) found that a new magnesium compound MgT could significantly increase the magnesium level in the brain and improve learning and memory function. Compared with other magnesium supplements, the molecular size of MgT is relatively small, and the absorption rate and retention rate are higher.

Sadia and colleagues studied the neurobehavior and biochemical effects of magnesium chloride(MgCl_2_), magnesium sulfate(Mg_2_SO_4_) and MgT supplementation in rats, and found that MgT is the most suitable magnesium salt for oral therapy, and 100 mg/kg is the most appropriate dose of magnesium (14). The possible mechanism of MgT on cognitive function is to enhance the cholinergic system by activating NMDA receptors, thereby increasing synaptic density, improving memory, and improving brain-related functions (13).

L-threonate (2R,3S)-2,3, 4-trihydroxybutanoate is an ascorbic acid metabolite that occurs naturally in the human body and is found in plasma, aqueous humor, urine, and brain (15). Rats ingestion of threonate salt alone without magnesium ions showed no significant change in memory ability, suggesting a synergistic effect between threonate and magnesium (13).

Biomarkers are biochemical indicators related to physiological and pathophysiological states of the body that can be monitored for changes (16), whose essence lies in “changes” (17). Due to the control of the homeostatic mechanisms, early small changes in blood are eliminated. Since urine is not part of the internal environment, in contrast to plasma, there is no homeostasis mechanism in urine. Urine collects waste from the whole body and reflects more sensitively the changes in the organism, which makes urine an ideal source of biomarkers (18).

Protein is essential for maintaining the normal physiological functions of cells and even the body, and the types and contents of proteins in body fluids will show different characteristics with the changes of the physiological state of the body (19). Proteins in urine contain a wealth of information that can reflect small changes in different systems and organs of the body. The main task of proteomics is to identify proteins present in cells, tissues or the body, analyze protein structure, molecular function and biological pathways in combination with various methods, and interpret the information carried by proteins (20). Urine proteomics is the science of studying the proteome in urine. It is one of the fields of biomarker research that has attracted much attention in recent years.

In this study, we established a rat model of short-term MgT intake to investigate the changes in the urinary proteome after short-term supplementation with MgT to explore the overall effects of the intake of MgT on the organism. This study innovatively establishes a method to study the effect of nutrients on the organism from the perspective of the urine proteome, which can reflect the role of the organism in a more comprehensive and systematic way and can hopefully provide information and clues about nutrient assessment, monitoring and prescription for clinical nutrition research and practice.

## 2 Materials and methods

### 2.1 Experimental materials

#### 2.1.1 Experimental consumables

Five-milliliter sterile syringes (BD), gavage needles (16-gage, 80 mm, curved needle), 1.5-ml/2-ml centrifuge tubes (Axygen, United States), 50-ml/15-ml centrifuge tubes (Corning, United States), 96-well cell culture plates (Corning, United States), 10 kD filters (Pall, United States), Oasis HLB solid phase extraction column (Waters, United States), 1-ml/200-μl/20-μl pipette tips (Axygen, United States), a BCA kit (Thermo Fisher Scientific, United States), high pH reverse peptide separation kit (Thermo Fisher Scientific, United States), and iRT (indexed retention time, BioGnosis, United Kingdom) were used.

#### 2.1.2 Experimental apparatus

Rat metabolic cages (Beijing Jiayuan Xingye Science and Technology Co., Ltd.), frozen high-speed centrifuge (Thermo Fisher Scientific, United States), vacuum concentrator (Thermo Fisher Scientific, United States), DK-S22 electric thermostatic water bath (Shanghai Jinghong Experimental Equipment Co., Ltd.), full-wavelength multifunctional enzyme labeling instrument (BMG Labtech, Germany), oscillator (Thermo Fisher Scientific, United States), TS100 constant temperature mixer (Hangzhou Ruicheng Instruments Co. BMG Labtech), electronic balance (METTLER TOLEDO, Switzerland), −80°C ultralow-temperature freezing refrigerator (Thermo Fisher Scientific, United States), EASY-nLC1200 Ultra High Performance Liquid Chromatography system (Thermo Fisher Scientific, United States), and Orbitrap Fusion Lumos Tribird Mass Spectrometer (Thermo Fisher Scientific, United States) were used.

#### 2.1.3 Experimental reagents

MgT was purchased from Shanghai Yuanye Biotechnology Co., CAS number is 778571–57-6, molecular formula is C8H14MgO10, purity is more than 95%. Trypsin Golden (Promega, United States), dithiothreitol (DTT, Sigma, Germany), iodoacetamide (IAA, Sigma, Germany), ammonium bicarbonate NH4HCO3 (Sigma, Germany), urea (Sigma, Germany), pure water (China Wahaha Company), mass spectrometry grade methanol (Thermo Fisher Scientific, United States), mass spectrometry grade acetonitrile (Thermo Fisher Scientific, United States), mass spectrometry grade purified water (Thermo Fisher Scientific, United States), Tris-base (Promega, United States), thiosulfate (Sigma, Germany), thiamine (Sigma, Germany), thiophene (Promega, United States), and thiourea (Sigma, Germany) were obtained.

#### 2.1.4 Analysis software

Proteome Discoverer (Version 2.1, Thermo Fisher Scientific, USA), Spectronaut Pulsar (Biognosys, United Kingdom), Ingenuity Pathway Analysis (Qiagen, Germany), R studio (Version 1.2.5001), Xftp 7, and Xshell 7 were utilized.

### 2.2 Experimental methods

#### 2.2.1 Animal modeling

In this study, 14-week-old rats were used to minimize the effects of growth and development during gavage. Five healthy Sprague–Dawley (SD) 9-week-old male rats (250 ± 20 g) were purchased fromVital River Laboratory (Animal Technology Co. Ltd., Beijing, China). The rats were kept in a standard environment (room temperature (22 ± 2)°C, humidity 65–70%) for 5 weeks and weighed 500 ± 20 g before starting the experiments, and all experimental operations followed the review and approval of the Ethics Committee of the School of Life Sciences, Beijing Normal University.

Slutsky and colleagues reported that 604 mg/kg/d MgT, i.e., 50 mg/kg/d elemental magnesium, is the minimum effective dose for memory enhancement in rat experiments (13). It has been reported that MgT is the most suitable magnesium salt for oral therapy, and 100 mg/kg is the most appropriate dose of magnesium (14). Therefore, the dose used in this study was 1,208 mg/kg/d MgT and 100 mg/kg/d elemental magnesium.

**Figure 1:**
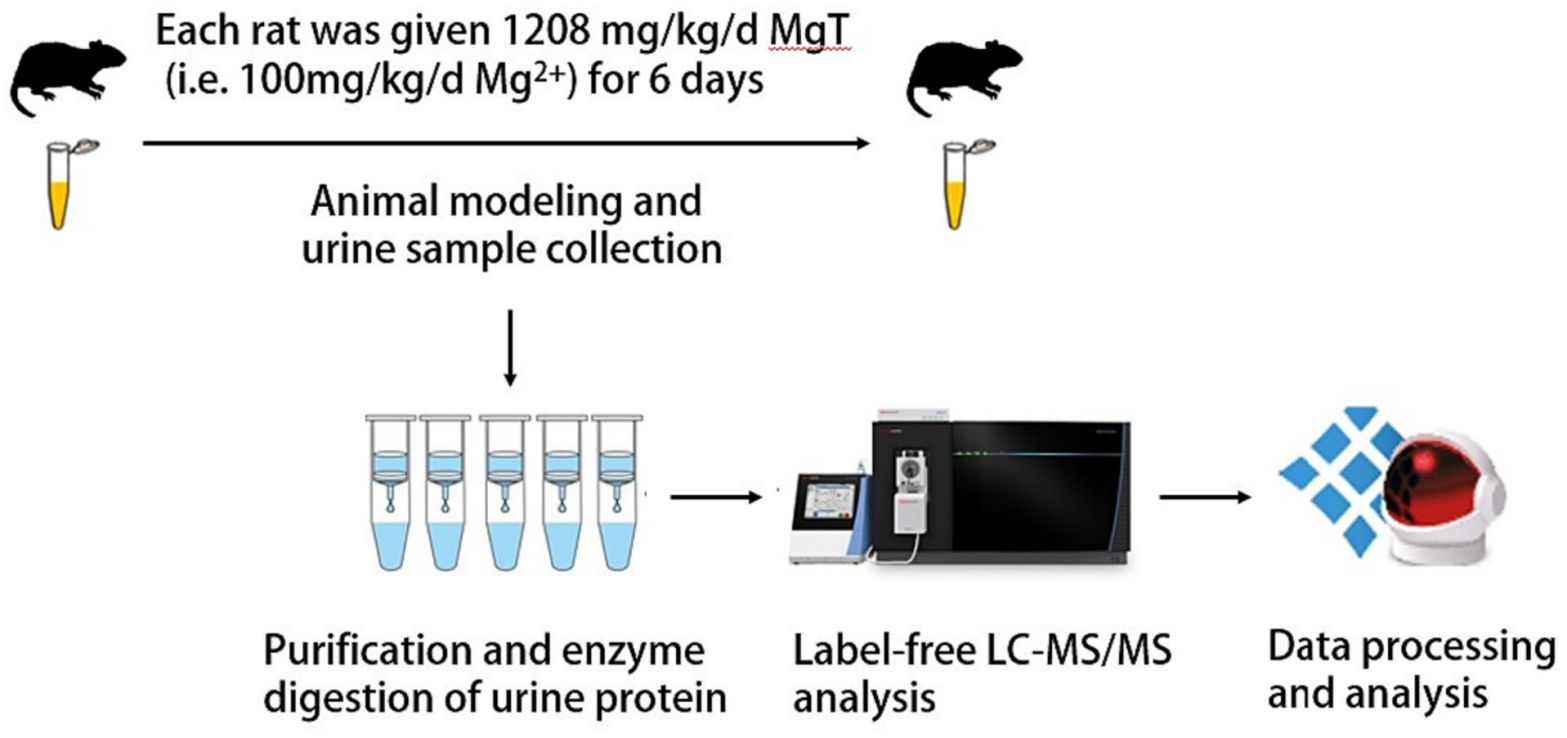
Flowcharts of the research.

24.16 g of MgT was dissolved in 200 mL of sterile water to configure a MgT solution. Each rat was oral gavaged with 5 mL of MgT solution once a day for 6 days. The first day of gavage was recorded as D1, and so on. Sampling time points were set on D0 and D6 so that each animal served as its own control and it could reduce the influence of individual factors. The sample collected on the day before gavage was the control group, recorded as D0. And the sample collected on the 6th day of gavage was the experimental group, recorded as D6 (Figure 1).

#### 2.2.2 Urine sample collection

One day before the start of MgT gavage (D0) and 6 days after MgT gavage (D6), each rat was individually placed in a metabolic cage at the same time of day, fasted and dehydrated for 12 h. Urine was collected overnight, and the urine samples were collected and placed in the freezer at −80°C for temporary storage.

#### 2.2.3 Urine sample processing

Two milliliters of urine sample was removed, thawed, and centrifuged at 4°C and 12,000 × g for 30 min. The supernatant was removed, and 1 M dithiothreitol (DTT, Sigma) storage solution (40 μL) was added to reach the working concentration of DTT (20 mM). The solution was mixed well and then heated in a metal bath at 37°C for 60 min and allowed to cool to room temperature.

Then, iodoacetamide (IAA, Sigma) storage solution (100 μL) was added to reach the working concentration of IAM, mixed well and then reacted for 45 min at room temperature protected from light. At the end of the reaction, the samples were transferred to new centrifuge tubes, mixed thoroughly with three times the volume of precooled anhydrous ethanol, and placed in a freezer at −20°C for 24 h to precipitate the proteins.

At the end of precipitation, the sample was centrifuged at 4°C for 30 min at 10,000 × g, the supernatant was discarded, the protein precipitate was dried, and 200 μL of 20 mM Tris solution was added to the protein precipitate to reconstitute it. After centrifugation, the supernatant was retained, and the protein concentration was determined by the Bradford method.

Using the filter-assisted sample preparation (FASP) method, urinary protein extracts were added to the filter membrane of a 10-kD ultrafiltration tube (Pall, Port Washington, NY, United States) and washed three times with 20 mM Tris solution. The protein was resolubilized by the addition of 30 mM Tris solution, and the protein was added in a proportional manner (urinary protein:trypsin = 50:1) to each sample. Trypsin (Trypsin Gold, Mass Spec Grade, Promega, Fitchburg, WI, United States) was used to digest proteins at 37°C for 16 h.

The digested filtrate was the peptide mixture. The collected peptide mixture was desalted by an Oasis HLB solid phase extraction column, dried under vacuum, and stored at −80°C. The peptide mixture was then extracted with a 0.1% peptide mixture. The lyophilized peptide powder was redissolved by adding 30 μL of 0.1% formic acid water, and then the peptide concentration was determined by using the BCA kit. The peptide concentration was diluted to 0.5 μg/μL, and 4 μL of each sample was removed as the mixed sample.

#### 2.2.4 LC–MS/MS analysis

All identification samples were added to a 100-fold dilution of iRT standard solution at a ratio of 20:1 sample:iRT, and the retention times were standardized. Data-independent acquisition (DIA) was performed on all samples, and each sample measurement was repeated 3 times, with 1-mix samples inserted after every 10 runs as a quality control. The 1-μg samples were separated using EASY-nLC1200 liquid chromatography (elution time: 90 min, gradient: mobile phase A: 0.1% formic acid, mobile phase B: 80% acetonitrile), the eluted peptides were entered into the Orbitrap Fusion Lumos Tribird mass spectrometer for analysis, and the corresponding raw files of the samples were generated.

#### 2.2.5 Data processing and analysis

The raw files collected in DIA mode were imported into Spectronaut software for analysis, and the highly reliable protein criterion was a peptide q value<0.01. The peak area quantification method was applied to quantify the proteins by applying the peak area of all fragment ion peaks of the secondary peptides.

Proteins containing two or more specific peptides were retained, and the contents of individual proteins identified in different groups of samples were compared to screen for differential proteins. The conditions for screening differential proteins were a *p* value <0.05 for paired t test analysis and fold change (FC) >5 or < 0.2.

Unsupervised cluster analysis, such as hierarchical cluster analysis (HCA) and principal component analysis (PCA), was performed using the wkomics platform,^1^ and functional enrichment of differential proteins was performed using the Database for Annotation, Visualization and Integrated Discovery (DAVID).^2^ Gene Ontology (GO) analysis was conducted to obtain the results from the 3 aspects of biological process, cellular localization and molecular function. Ingenuity Pathway Analysis (IPA) was performed for the identified differential proteins, and a search was conducted for the differential proteins and related pathways based on the PubMed database.^3^

The mass spectrometry proteomics data have been deposited to the ProteomeXchange Consortium^4^ via the iProX partner repository with the dataset identifier PXD046121 (21, 22).

#### 2.2.6 Randomized grouping analysis

A total of 10 samples before (*n* = 5) and after (*n* = 5) gavage were randomly divided into two groups, and the mean of the number of differential proteins in all randomized combinations was calculated according to the screening conditions of *p* value <0.05 and fold change (FC) >5 or < 0.2.

## 3 Results and discussion

### 3.1 Analysis of urinary proteome changes before and after ingestion of MgT

**Figure 2:**
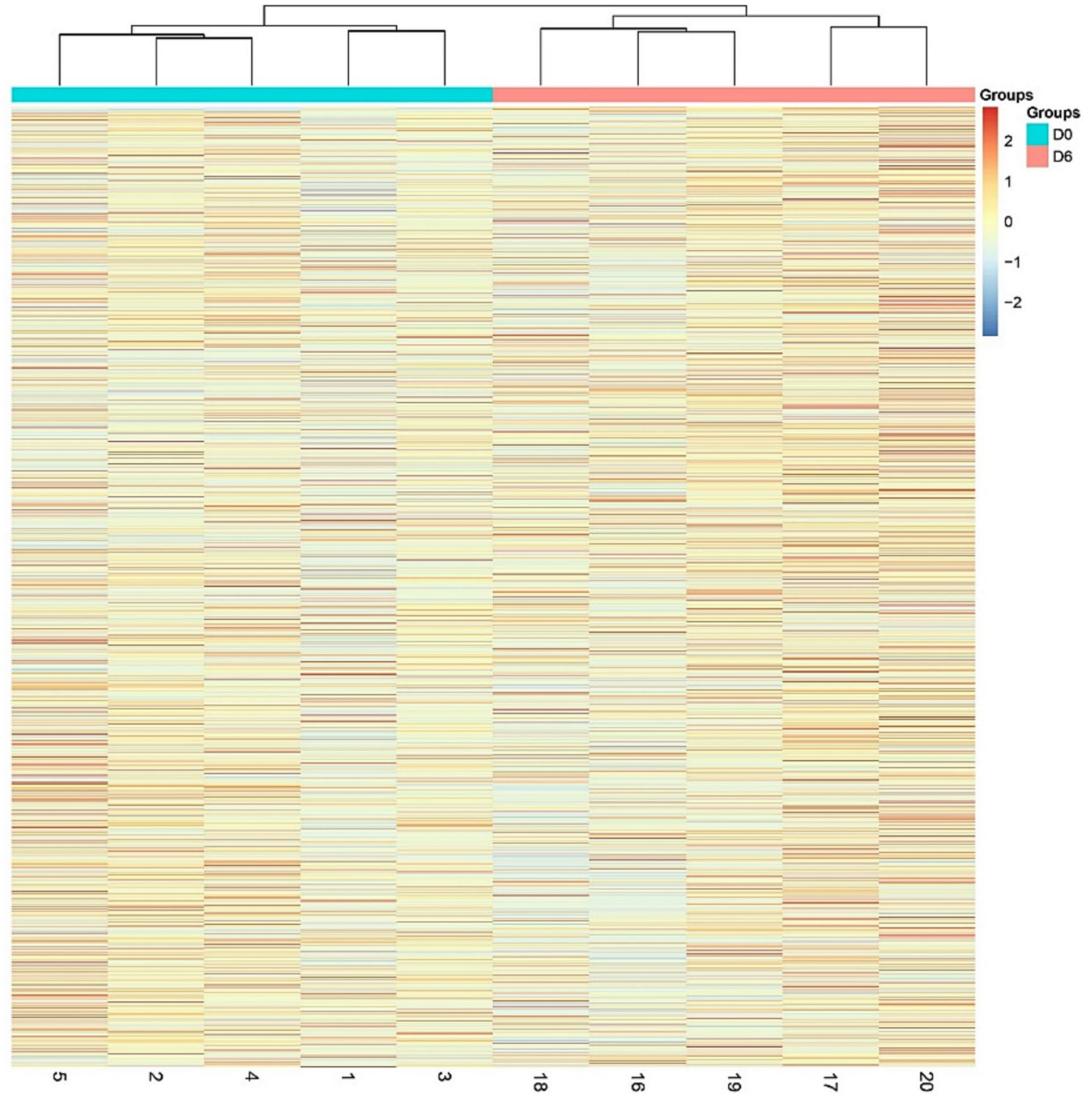
Hierarchical cluster analysis (HCA) of the total proteins in the samples.

**Figure 3:**
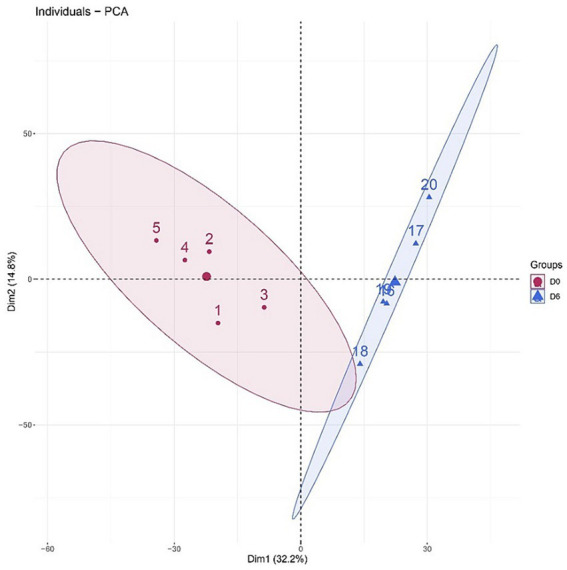
Principal component analysis (PCA) of the total proteins in the samples.

A total of 1766 proteins were identified in all samples (meeting unique peptides>1 and FDR < 1%). The results of unsupervised cluster analysis and principal component analysis (PCA) showed that the two groups of samples could be well distinguished, indicating significant changes in the rat urine proteome after continuous gavage of MgT for 6 days, as shown in Figures 2, 3.

### 3.2 Randomized grouping test results

We used a randomized grouping statistical analysis strategy, which is suitable for the study of proteomes with limited sample sizes and for determining whether the differences between two groups are randomly generated (23).

To determine the likelihood of random generation of the identified differential proteins, we validated the random grouping of total proteins identified from two groups of 10 samples, applying the same criteria for the screening of differential proteins, that is, FC ≥ 5 or ≤ 0.2 and *p* < 0.05, and performing 126 random groupings yielded an average of 20.53 differential proteins, with a proportion of randomly identified proteins of 8.18%, indicating that at least 91.82% of the differential proteins were not due to randomness.

The results of the randomized grouping test are shown in Table 1. The probability that the 251 differential proteins we identified by screening were randomly generated is low, and the results show that these differential proteins are indeed associated with short-term intake of MgT.

**Table 1:** Randomized grouping results.

Screening criteria	Total number of random combinations	Average number of proteins with false random combinations	Ratio (average numbers of proteins with false random combinations/number of correctly identified differential proteins)
FC ≥ 5 or ≤ 0.2 *p* < 0.05	126	20.53	8.18%

### 3.3 Differentially expressed protein function annotation

The missing values were replaced with 0. The pregavage samples of rats with MgT were compared with the samples on Day 6 of gavage, and 251 differential proteins were identified under the screening conditions of FC ≥ 5 or ≤ 0.2, and *p* < 0.05, as detailed in the Supplementary Table.

Among them, 63 differential proteins showed significant changes with the largest fold changes (FC = 0 or ∞), such as solute carrier family 21 (SLC21), angiotensin-converting enzyme, collagen type X alpha 1 chain, and heat shock 70 kDa. The fold change of 58 differential proteins, including collagen type X alpha 1 chain, heat shock 70 kDa protein, integrin alpha 3, and protein-tyrosine-phosphatase (PTP), was zero, which means that they were detected in the urinary proteome of rats before gavage but were so low as to be nondetectable in the urinary proteome of rats after 6 days of gavage. Four differential proteins, including PDZ and LIM domain protein 1, had a fold change of ∞, i.e., the protein was undetectable in the rat urine proteome before gavage but was detectable in the rat urine proteome after 6 days of gavage.

Functional annotation and searching of these differential proteins using the UniProt database and PubMed database revealed that many of the significantly changed differential proteins were directly or indirectly related to magnesium, as shown in Table 2.

**Table 2:** Differential proteins showing significant changes (FC = 0 or ∞, *p* < 0.05).

Protein accession	Gene	Fold change	*p* Value	Citations
A0A0A0MXT4	Slc21a4;Slco1a3	0	0.022589	(24, 25)
A0A0G2JUN7	Txnrd1	0	0.040212	(26)
A0A0G2JX26	Slc17a3	0	0.028506	(24, 25)
A0A0G2JXU8	Ace2	0	0.016671	(27)
A0A0G2K2B6	Tpmt	0	0.035571	(26)
A0A0G2K2V7	Yes1	0	0.037625	(28)
A0A0G2K4K4	Slc12a1	0	0.043945	(24, 25)
A0A0G2K595	Slc23a1	0	0.006815	(24, 25)
A0A0G2K7A5	Col10a1	0	0.043308	(29)
A0A0G2K931	Psat1	0	0.04298	
A0A0G2QC16	Aox4	0	0.004368	(30)
A0A0H2UHJ0	Shbg	0	0.005177	
A2RUW1	Tollip	0	0.016499	(31)
B0BNL6	Arrdc1	0	0.039319	(32)
B2GV72	Cbr3	0	0.006854	(33)
D3Z9E5	Slc5a8	0	0.010938	(24, 25)
D3ZBE9	Adgrl2	0	0.04395	
D3ZC55	Hspa12a	0	0.011409	(34)
D3ZCG9	Itga3	0	0.018798	(35, 36)
D4AC85	Qpct	0	0.0438	
F1LY81	Rhpn1	0	0.030016	
G3V637	Stxbp2	0	0.001708	
G3V6Q6	Gna11	0	0.00309	
G3V6Y3	Kif12	0	0.047406	
G3V7Q8	Prss3b;Try3	0	0.004184	
G3V847	Slc6a18	0	0.027469	(24, 25)
M0R7M5	Ig-like domain-containing protein	0	0.041422	
M0RC99	Rab5a	0	0.002108	(37)
O35142	Copb2	0	0.01584	
P04897	Gnai2	0	0.000302	
P46720	Slco1a1	0	0.037505	(24, 25)
P50398	Gdi1	0	0.000713	(37)
P51907	Slc1a1	0	0.025198	(24, 25)
P53790	Slc5a1	0	0.023289	(24, 25)
P54313	Gnb2	0	7.43E-06	
P63095	Gnas	0	5.84E-06	
P63322	Rala	0	0.002839	
Q04970	Nras	0	0.002173	(38)
Q3T1K5	Capza2	0	0.000229	
Q3ZAV1	Slc22a12	0	0.03797	(24, 25)
Q5BJN1	Stard10	0	0.018192	
Q5BJU0	Rras2	0	0.017365	
Q5I0E9	Slc47a1	0	0.004751	(24, 25, 39)
Q5RJR2	Twf1	0	0.012608	
Q5RKI7	Slc7a13	0	0.038814	(24, 25)
Q5U355	Itfg1	0	0.0471	(35, 36)
Q62797	Ptpro	0	0.012681	(40)
Q63355	Myo1c	0	1.33E-05	(41)
Q63357	Myo1d	0	0.00077	(41)
Q63942	Rab3d	0	0.048246	(37)
Q66HG3	Cndp1	0	0.014954	
Q6IRE4	Tsg101	0	0.000179	(42)
Q6P9U0	Serpinb6a	0	0.014935	(42)
Q8CGS4	Chmp3	0	0.000107	
Q8R3Z7	Ehd4	0	0.006875	
Q8R431	Mgll	0	0.018125	
Q99376	Tfrc	0	0.003243	
Q9ES53	Ufd1	0	6.81E-06	
D4AA77	Plxnd1	∞	0.041948	
F1M1Q3	Adam33	∞	0.000265	
G3V714	Scg5	∞	0.033025	(43)
P52944	Pdlim1	∞	0.019866	(44)
Q6IMK4	Svs1	∞	0.00546	

According to the literature, the expression levels or activities of many differential proteins are positively or negatively correlated with magnesium levels. For instance, angiotensin-converting enzyme is a peptidase involved in the formation of angiotensin II and the inactivation of bradykinin. In one study, women with gestational hypertension had elevated angiotensin-converting enzyme activity before treatment with magnesium sulfate and lower levels during treatment (27). Magnesium intake may influence blood pressure regulatory mechanisms.

Moreover, in *in vitro* culture experiments, magnesium promoted bone regeneration by promoting osteoblast type X collagen and VEGF production in bone tissue (29). Magnesium supplementation inhibits the upregulated expression of HSP70 and HSP70 mRNA *in vivo* (45). Magnesium promote cell proliferation mediated by integrin α2 and integrin α3 (35). Magnesium ions and zinc ions activate and inhibit protein tyrosine phosphatase 1B (PTP1B), respectively (40). Magnesium inhibits the collagen-cleaving activity of the 41-kDa serine-dependent protease, and calcium protects the protease from this inhibition (46). Magnesium sulfate inhibits LPS-macrophage binding by decreasing CD14 expression, and these mechanisms may involve the activation of serine proteases (47).

Many processes in which differential proteins function require the involvement of magnesium. For example, magnesium ions play an important role in NADPH oxidase function (33). Of the differential proteins showing significant changes with or without magnesium, at least five differential proteins were associated with ATPase and ADP/ATP binding, and at least five were associated with GTPase and GDP/GTP binding. Magnesium ions have the ability to form chelates with ATP and GTP, and many ATPases and GTPases are magnesium-dependent enzymes (48). In addition, Rab5a and NRAS were two differential proteins that exhibited significant changes. Magnesium ions interact with the GTP form and GDP form of Rab5 and Rab7 (37). Magnesium ions play a bridging role in the interaction of ligands with NRAS, favoring the association of GDP, GTP and GNP with NRAS (38).

A number of differential proteins have been associated with the transport of magnesium ions. For example, a total of 14 differential proteins belong to the solute carrier family, and the solute carrier family members 1 and 2 (SLC41A1 and SLC41A2) channels of this family are capable of transporting magnesium ions (24).

### 3.4 Molecular functional analysis of differentially expressed protein enrichment

**Figure 4:**
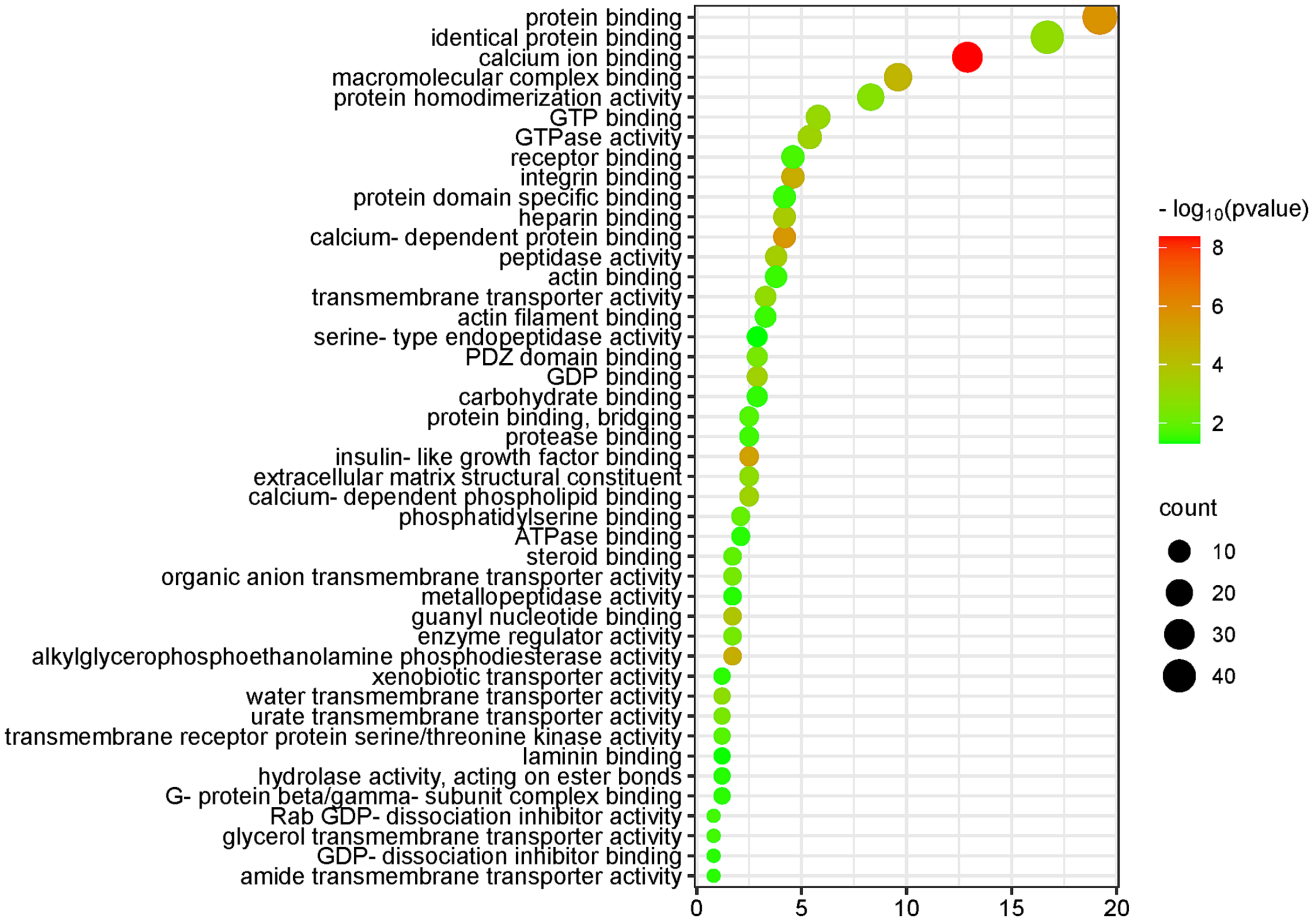
Molecular function (MF) enrichment analysis of the differential proteins (DAVID database, GO analysis).

Using the DAVID database to enrich and analyze the molecular functions of 251 differential proteins (screening conditions FC ≥ 5 or ≤ 0.2 and *p* < 0.05), 45 molecular functions, such as calcium binding, protein binding, insulin-like growth factor binding, integrin binding, GTP binding, PDZ domain binding, transmembrane transport, enzyme regulation, and enzyme activity, were enriched (*p* < 0.05). The molecular functions, protein numbers and *p* values are shown in Figure 4.

The five molecular functions with the highest significance were calcium binding, protein binding, calcium-dependent protein binding, insulin-like growth factor binding, and integrin binding.

The molecular functions of calcium ion binding, calcium-dependent protein binding, and calcium-dependent phospholipid binding, which are closely related to calcium ions, were enriched in 35 differential proteins. The molecular function of protein binding was enriched in 46 differential proteins. Magnesium ions have the ability to compete with calcium ions for binding sites on proteins and membranes, and magnesium contributes to the maintenance of a low resting intracellular concentration of free calcium ions, which is important in many cellular functions (49, 50).

The molecular function of insulin-like growth factor binding was enriched in six differential proteins. According to the literature, insulin-like growth factor 1 (IGF-1) plays a role in cellular magnesium metabolism and can enhance insulin sensitivity (51). Magnesium exerts insulin sensitization through autophosphorylation of the insulin receptor and regulation of tyrosine kinase activity on the receptor (52). Magnesium is an essential cofactor for several enzymes involved in carbohydrate metabolism (53). Magnesium deficiency is associated with an increased incidence of diabetes mellitus (54).

Integrin binding is a molecular function that was enriched in 11 differential proteins. Magnesium ions promote cell attachment through binding interactions between the integrin family and FAK-related signaling pathways (36).

Magnesium can regulate ion transport via pumps, carriers and channels (55). Many of the molecular functions enriched by differential proteins are related to transmembrane transport, including transmembrane transporter protein activity, water transmembrane transporter protein activity, urate transmembrane transporter protein activity, organic anion transmembrane transporter protein activity, transmembrane receptor protein serine/threonine kinase activity, glycerol transmembrane transporter protein activity, heterotrimeric transporter activity, and amide transmembrane transporter activity, among other molecular functions.

Water transmembrane transporter activity was enriched in three differential proteins, and the fold change of aquaporin-1 (AQP-1), a differentially expressed protein, was 0.005. According to the literature, magnesium ions regulate the expression of the water channel proteins AQP-3 and AQP-4 (56).

The molecular function of PDZ domain binding was enriched in eight differential proteins. It has been reported in the literature that PDZ domain containing ring finger 3 (PDZRN3) is able to regulate the magnesium regulator claudin-16 localized in renal tubular epithelial cells (44).

Enzyme-related molecular functions enriched in differential proteins included alkylglycerophosphoethanolamine phosphodiesterase activity, peptidase activity, GTPase activity, enzyme regulatory activity, transmembrane receptor protein serine/threonine kinase activity, protease binding, hydrolase activity, metalloproteinase activity, and serine-type endopeptidase activity.

At least 60 differential proteins belong to the class of enzymes and are closely related to the synthesis of sugars, proteins, nucleic acids and other molecules; signal transduction; and energy metabolism processes.

Magnesium is a cofactor in hundreds of enzymatic reactions, including those mediated by phosphotransferases and hydrolases such as ATPase (48). Magnesium affects enzyme activity by binding to a ligand, binding to the active site of an enzyme, causing a conformational change in the catalytic process, promoting the aggregation of multienzyme complexes, or other mechanisms (48). Magnesium-dependent enzymes include Na/K-ATPase, hexokinase, creatine kinase, protein kinase, aldehyde dehydrogenase, and cyclases (50).

These enzyme systems regulate a variety of biochemical reactions in the body, including protein synthesis, muscle and neurotransmission, neuromuscular transmission, signal transduction, blood glucose control, and blood pressure regulation. Magnesium is required for protein and nucleic acid synthesis, the cell cycle, cytoskeletal and mitochondrial integrity, energy production, and binding of substances to the plasma membrane (5).

Differential proteins enriched molecular functions such as GTP binding, GDP binding, GTPase activity, Rab GDP-dissociation inhibitor activity, G-protein beta/gamma-subunit complex binding, GDP-dissociation inhibitor binding and other molecular functions. Magnesium ions have the ability to form chelates with the important anionic ligand GTP, and GTPases are magnesium-dependent enzymes (48). Magnesium ions play a role in the G protein-coupled receptor signaling pathway (57).

The two molecular functions of actin binding and actin filament binding were enriched in a total of 14 differential proteins. Magnesium regulates actin binding and ADP release in the myosin motor domain (58).

### 3.5 Analysis of the biological processes with differentially expressed protein enrichment

The DAVID database and IPA database were used to enrich and analyze the biological processes and biological pathways of 251 differential proteins (the screening conditions were FC ≥ 5 or ≤ 0.2, and *p* < 0.05).

**Figure 5:**
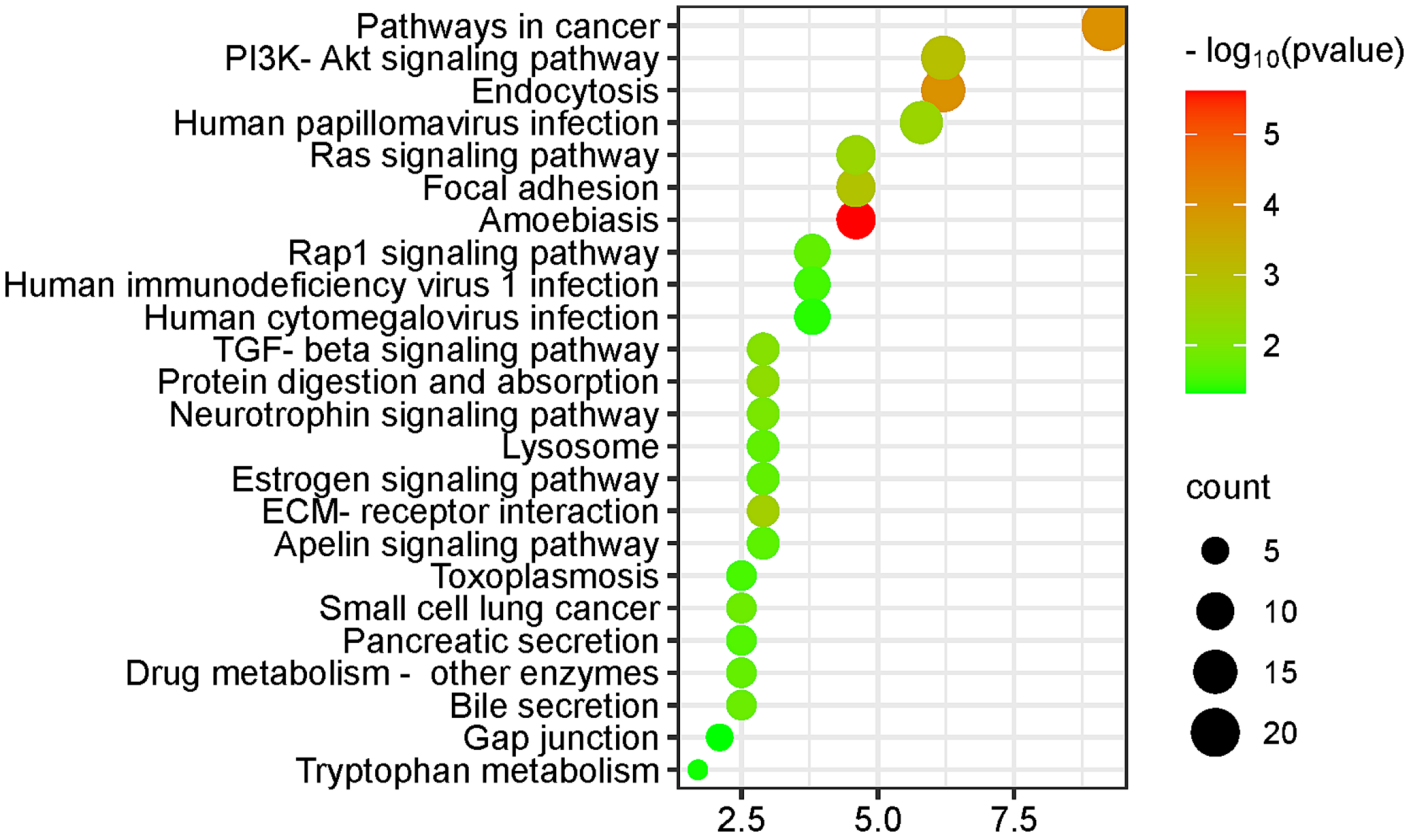
KEGG pathway enrichment analysis of the differential proteins (DAVID database, GO analysis).

The KEGG pathway results for differentially expressed protein enrichment are shown in Figure 5; the biological process (BP) results are shown in Supplementary Figure S2; and the IPA results are shown in Supplementary Figure S1.

The differential proteins enriched many KEGG pathways related to cancer and immunity, including the pathway of cancer, TGF-β signaling pathway, extracellular matrix (ECM) receptor interaction pathway, and small cell lung cancer. Biological processes that were enriched included regulation of cell migration and regulation of cell death. Magnesium levels have been reported to be associated with cancer risk and treatment outcome (59).

In addition, magnesium ions have been reported to enhance the transfer of human oncovirus E2 protein from a nonspecific binding site to a specific binding site (60). High levels of magnesium ions can act as a therapeutic agent for chronic amoebiasis (61).

Biological processes such as leukocyte migration and the adaptive immune response are associated with the immune response. Magnesium plays a role in immune function, and magnesium ions are involved in macrophage activation, bactericidal activity of the granulocyte oxidative burst, lymphocyte proliferation, and endotoxin binding to monocytes (6).

The PI3K-Akt signaling pathway was enriched in 15 differential proteins. The PI3K-Akt signaling pathway is the classical response to insulin signaling, which is mediated by serine or threonine phosphorylation of a series of downstream substrates. The key genes involved are phosphatidylinositol 3-kinase (PI3K) and AKT/protein kinase b. There is sufficient evidence that magnesium activate the PI3K-Akt signaling pathway (62, 63).

At the same time, the biological process of regulation of glucose metabolic process was enriched in three differential proteins, and four differential proteins were associated with the positive regulation of insulin secretion, as detailed in the Supplementary Figure S1. According to the latest guidelines from the Association for Magnesium Research, magnesium supplementation can benefit diabetic individuals in different ways: insulin sensitization, calcium antagonism, blood pressure regulation and endothelial stabilization (64).

The neurotrophin signaling pathway, a cell signaling mechanism that plays a key role in the development, plasticity and repair of the nervous system, was enriched in seven differential proteins. The biological processes which the differential proteins enriched included learning or memory, axon guidance, axon regeneration, positive regulation of neuron projection development, and nervous system development.

Magnesium is a modulator of glutamatergic N-methyl-D-aspartic acid (NMDA) receptor affinity and can regulate nerve-muscle junction excitability, which may be related to the neurobiological characteristics of mood disorders and psychiatric disorders. Animal experiments have revealed that magnesium can improve hippocampal synaptic plasticity and learning memory in rats (65). Magnesium has the potential to be used as an adjunctive drug in the treatment of depression, anxiety, attention deficit hyperactivity disorder and other psychiatric disorders and Alzheimer’s disease (43, 66).

The KEGG pathway was enriched for seven differential proteins in the apelin signaling pathway. The apelin signaling pathway plays an important role in the maintenance of endothelial homeostasis and immunomodulation as well as in the protection of lipids from oxidation, and magnesium sulfate can be used to regulate serum apelin levels (67).

Differential proteins enriched biological processes including angiogenesis, blood coagulation, and heart development. Dietary magnesium deficiency plays an important role in the pathogenesis of ischemic heart disease, congestive heart failure, sudden cardiac death, cardiac arrhythmias, diabetic vascular complications, preeclampsia/eclampsia, and hypertension (6). Several studies have also shown the beneficial effects of magnesium supplementation in the treatment of the above diseases.

The differential proteins enriched many biological processes related to transmembrane transport, including protein transport, organic anion transport, sodium ion transport, extracellular transport, water transport, and responses to various ions, such as mercury, iron, calcium, cadmium, zinc, and copper. Magnesium ions often regulate ion transport through pumps, carriers, and channels (55), which in turn can regulate signaling and the cytoplasmic concentration of ions such as calcium and potassium.

The adenylate cyclase-activating G-protein coupled receptor signaling pathway was enriched in six differential proteins, and the biological process of small GTPase-mediated signaling was enriched in five differential proteins. Magnesium ions have been reported to directly activate adenylate cyclase (48), and to play a role in the G protein-coupled receptor signaling pathway (57).

Differentially expressed protein enrichment in biological processes included bone resorption, osteoblast differentiation, osteoclast proliferation, osteoclast differentiation, ossification, and processes related to bone development and bone metabolism. The literature suggests a causal relationship between dietary intake of magnesium and maintenance of normal bone.

The biological process of lung development was enriched for seven differential proteins. The literature suggests that dietary magnesium intake is associated with lung function, airway responsiveness and respiratory symptoms (68).

The estrogen signaling pathway was enriched for seven differential proteins (see Figure 5). Many hormones, including sex steroids, have been reported to affect magnesium homeostasis (69).

### 3.6 Discussion

In this study, a number of methods were used to minimize the influence of irrelevant factors. First, using the same group of animals, sampling time points were set on D0 and D6 so that each animal served as its own control and it could reduce the influence of individual factors. Second, middle-aged rats aged 14 weeks were treated by intragastric administration for 6 days to reduce the effects of growth, development and aging. Third, only the variable of intake of MgT was set, while other feeding conditions and urine collection conditions remained consistent, and the influence of unrelated factors was reduced. Fourth, strict screening conditions were set, and the differential proteins screened by FC > 5 or 0.2 and *p* < 0.05 showed significant changes before and after. Proteins that fluctuate slightly due to accidental errors can also be excluded to a certain extent.

However, this study also has some shortcomings, which we will discuss here. First, due to limited resources, this study only set up a treatment group with one dose (100 mg/kg d magnesium) and intragastric administration for 6 days to conduct an exploratory study.

Second, we use MgT as a magnesium supplement but magnesium related content is mainly described in the results and discussion section. We cannot find out whether differential proteins, biological processes, molecular functions and pathways of differential protein enrichment are related to L-threonate. On the one hand, it may be that there are few studies and publications related to L-threonate. On the other hand, it may be that the effect of L-threonate is relatively small, which is masked by magnesium.

Third, this study can only obtain results based on comparing changes in urine proteome before and after intake of MgT. The mechanism of action of MgT and the specific processes in the body still need to be further explored by other researchers.

## 4 Prospective

Micronutrients (MNs) include minerals and vitamins, and mineral elements can be categorized into macrominerals and microminerals according to human needs. Unlike macronutrients such as carbohydrates, proteins, and fats, micronutrients are required by the human body in very small amounts (70). Although micronutrient requirements are small, micronutrients play an important role in regulating and maintaining various physiological functions in the human body. Micronutrients are essential for cellular function, antioxidant defense and enzymatic reactions; and are involved in many processes, such as immune function, bone health, coagulation, and neurological function (71, 72).

Several vitamins (including vitamins A, B6, B12, C, D, E, and folic acid) and trace mineral elements (including zinc, iron, selenium, magnesium, and copper) have been reported to play important and complementary roles in supporting the innate and adaptive immune systems. Micronutrient imbalances can negatively impact immune function and can reduce resistance to infection (73). Epidemiologic studies have shown that intake levels of trace minerals such as magnesium, iron, zinc, and copper are associated with the incidence of coronary artery disease (74).

Much of the literature suggests that magnesium can play an important therapeutic and preventive role in a wide range of diseases, such as diabetes, osteoporosis, bronchial asthma, preeclampsia, migraines and cardiovascular disease (6), and has an adjunctive effect on the efficacy of certain medications. Deficiencies or excesses of various types of micronutrients are closely associated with various diseases.

It is evident that micronutrients are essential for good health, and deficiencies or excesses of micronutrients may lead to dysfunctions in the body, affecting signaling disorders, cellular processes and organ dysfunctions, which can lead to the development of pathological conditions (75). The impact of nutrient supplementation on the prevention and treatment of diseases should not be ignored, and the study of the role of nutrient intake on the organism is important for disease monitoring, assessment of the effectiveness of treatment and prognosis. Therefore, it is meaningful to explore biomarkers related to nutrient intake.

Previous nutritional studies have usually been limited to focusing on the content or intake of the nutrient itself. The most common method is to measure the concentration of a particular nutrient in a sample of body fluid. However, for some nutrients, extrapolating the level of body stores by directly assessing specific levels of a particular nutrient in body fluids is inaccurate and often invasive. In the case of magnesium, for example, magnesium levels of serum have little correlation with systemic magnesium levels (8). Some researchers have also chosen to use metabolites of foods as markers of certain nutrient intakes (76). Some researchers use subjective dietary recall for dietary assessment (77). However, this type of intake assessment is not very accurate and only reflects intake, not the body’s reflection of the nutrient.

Urinary proteins originate from the body and can reflect small changes in the body and even have the potential to reflect the corresponding biological processes, signaling pathways and molecular mechanisms, which can reflect changes in the physiological state of the organism in a more comprehensive and systematic manner. To the author’s knowledge, no study has yet researched the overall effect of nutrients on the organism from a urinary proteomic perspective.

Our study establishes a novel approach to study nutrients, focusing on the urine proteome, to investigate how short-term supplementation with moderate amounts of nutrients affect the body. It provides a new research perspective in nutrition, which is groundbreaking in the field of nutrition, and has the potential to provide information and clues about nutrient assessment and monitoring for clinical nutrition research and practice.

The results of the study illustrate that the urinary proteome of rats can reveal changes in proteins and biological functions related to magnesium ions after short-term intake of MgT. In conclusion, short-term supplementation with MgT can have an effect on the organism, and the urinary proteome reflects this change more comprehensively and systematically.

## Data availability statement

The original contributions presented in the study are publicly available. The mass spectrometry proteomics data are available at the ProteomeXchange Consortium via the iProX partner with the dataset identifier PXD046121.

## Ethics statement

The animal study was approved by Beijing Normal University Animal Welfare and Ethics Committee. The study was conducted in accordance with the local legislation and institutional requirements.

## Author contributions

ZS: Writing – original draft. MY: Writing – original draft. HW: Writing – original draft. YL: Resources, Writing – original draft. YG: Writing – review & editing.

## Footnotes

^1^ https://omicsolution.org/wkomics/main/

^2^ https://david.ncifcrf.gov/

^3^ https://pubmed.ncbi.nlm.nih.gov/

^4^ http://proteomecentral.proteomexchange.org

## References

[1] Ebel H Günther T. Magnesium metabolism: a review. J Clin Chem Clin Biochem. (1980) 18:257–70. doi: 10.1515/cclm.1980.18.5.257 7000968

[2] Caspi R Altman T Dreher K Fulcher CA Subhraveti P Keseler IM. The MetaCyc database of metabolic pathways and enzymes and the BioCyc collection of pathway/genome databases. Nucleic Acids Res. (2012) 40:D742–53. doi: 10.1093/nar/gkr1014, PMID: 22102576 PMC3245006

[3] Bairoch A. The ENZYME database in 2000. Nucleic Acids Res. (2000) 28:304–5. doi: 10.1093/nar/28.1.304, PMID: 10592255 PMC102465

[4] de Baaij JHF Hoenderop JGJ Bindels RJM. Magnesium in man: implications for health and disease. Physiol Rev. (2015) 95:1–46. doi: 10.1152/physrev.00012.2014 25540137

[5] Gröber U Schmidt J Kisters K. Magnesium in prevention and therapy. Nutrients. (2015) 7:8199–226. doi: 10.3390/nu7095388, PMID: 26404370 PMC4586582

[6] Al Alawi AM Majoni SW Falhammar H. Magnesium and human health: perspectives and research directions. Int J Endocrinol. (2018) 2018:9041694. doi: 10.1155/2018/9041694 29849626 PMC5926493

[7] Song Y Ridker PM Manson JE Cook NR Buring JE Liu S. Magnesium intake, C-reactive protein, and the prevalence of metabolic syndrome in middle-aged and older U.S. women. Diabetes Care. (2005) 28:1438–44. doi: 10.2337/diacare.28.6.1438, PMID: 15920065

[8] Ismail Y Ismail AA Ismail AAA. The underestimated problem of using serum magnesium measurements to exclude magnesium deficiency in adults; a health warning is needed for “normal” results. Clin Chem Lab Med. (2010) 48:323–7. doi: 10.1515/CCLM.2010.077, PMID: 20170394

[9] Noronha JL Matuschak GM. Magnesium in critical illness: metabolism, assessment, and treatment. Intensive Care Med. (2002) 28:667–79. doi: 10.1007/s00134-002-1281-y 12107669

[10] National Health Commission of the People’s Republic of China. Dietary guidelines for Chinese residents. Beijing: Standards Press of China (2017).

[11] Marx A Neutra RR. Magnesium in drinking water and ischemic heart disease. Epidemiol Rev. (1997) 19:258–72. doi: 10.1093/oxfordjournals.epirev.a017957 9494787

[12] Olza J Aranceta-Bartrina J González-Gross M Ortega R Serra-Majem L Varela-Moreiras G. Reported dietary intake, disparity between the reported consumption and the level needed for adequacy and food sources of calcium, phosphorus, magnesium and vitamin D in the Spanish population: findings from the ANIBES study. Nutrients. (2017) 9:168. doi: 10.3390/nu9020168, PMID: 28230782 PMC5331599

[13] Slutsky I Abumaria N Wu L-J Huang C Zhang L Li B. Enhancement of learning and memory by elevating brain magnesium. Neuron. (2010) 65:165–77. doi: 10.1016/j.neuron.2009.12.026, PMID: 20152124

[14] Sadir S Tabassum S Emad S. Neurobehavioral and biochemical effects of magnesium chloride (MgCl2), magnesium sulphate (MgSO4) and magnesium-L-threonate (MgT) supplementation in rats: a dose dependent comparative study. Pak J Pharm Sci. (2019) 32:277–83. 30829204

[15] Sun Q Weinger JG Mao F Liu G. Regulation of structural and functional synapse density by L-threonate through modulation of intraneuronal magnesium concentration. Neuropharmacology. (2016) 108:426–39. doi: 10.1016/j.neuropharm.2016.05.006, PMID: 27178134

[16] Biomarkers Definitions Working Group. Biomarkers and surrogate endpoints: preferred definitions and conceptual framework. Clin Pharmacol Ther. (2001) 69:89–95. doi: 10.1067/mcp.2001.113989 11240971

[17] Gao Y. Urine-an untapped goldmine for biomarker discovery? Sci China Life Sci. (2013) 56:1145–6. doi: 10.1007/s11427-013-4574-1, PMID: 24271956

[18] Gao Y. On research and translation of urinary biomarkers. Adv Exp Med Biol. (2021) 1306:101–8. doi: 10.1007/978-3-030-63908-2_7 33959908

[19] Zhao M Yang Y Guo Z Shao C Sun H Zhang Y. A comparative proteomics analysis of five body fluids: plasma, urine, cerebrospinal fluid, amniotic fluid, and saliva. PROTEOMICS – Clinic. App. (2018) 12:e1800008. doi: 10.1002/prca.201800008, PMID: 29781159

[20] Wu J Gao Y. Physiological conditions can be reflected in human urine proteome and metabolome. Expert Rev Proteomics. (2015) 12:623–36. doi: 10.1586/14789450.2015.1094380, PMID: 26472227

[21] Ma J Chen T Wu S Yang C Bai M Shu K. iProX: an integrated proteome resource. Nucleic Acids Res. (2019) 47:D1211–7. doi: 10.1093/nar/gky869, PMID: 30252093 PMC6323926

[22] Chen T Ma J Liu Y Chen Z Xiao N Lu Y. iProX in 2021: connecting proteomics data sharing with big data. Nucleic Acids Res. (2021) 50:D1522–7. doi: 10.1093/nar/gkab1081 PMC8728291 34871441

[23] Meng W. Randomized grouping statistical analysis in clinical omics biomarker discovery. MOJ Proteomics Bioinform. (2020) 9:73–5.

[24] Ferrè S Hoenderop JGJ Bindels RJM. Insight into renal Mg2+ transporters. Curr Opin Nephrol Hypertens. (2011) 20:169–76. doi: 10.1097/MNH.0b013e3283435ee4, PMID: 21191290

[25] Mastrototaro L Smorodchenko A Aschenbach JR Kolisek M Sponder G. Solute carrier 41A3 encodes for a mitochondrial mg(2+) efflux system. Sci Rep. (2016) 6:27999. doi: 10.1038/srep27999, PMID: 27302215 PMC4908428

[26] Richter AS Peter E Rothbart M Schlicke H Toivola J Rintamäki E. Posttranslational influence of NADPH-dependent thioredoxin reductase C on enzymes in tetrapyrrole synthesis. Plant Physiol. (2013) 162:63–73. doi: 10.1104/pp.113.217141, PMID: 23569108 PMC3641230

[27] Fuentes A Goldkrand JW. Angiotensin-converting enzyme activity in hypertensive subjects after magnesium sulfate therapy. Am J Obstet Gynecol. (1987) 156:1375–9. doi: 10.1016/0002-9378(87)90003-2, PMID: 3035926

[28] Zou Z-G Rios FJ Montezano AC Touyz RM. TRPM7, magnesium, and signaling. Int J Mol Sci. (2019) 20:1877. doi: 10.3390/ijms20081877, PMID: 30995736 PMC6515203

[29] Yoshizawa S Brown A Barchowsky A Sfeir C. Role of magnesium ions on osteogenic response in bone marrow stromal cells. Connect Tissue Res. (2014) 55:155–9. doi: 10.3109/03008207.2014.923877, PMID: 25158202

[30] Weiner H Takahashi K. Role of magnesium and calcium ions in the regulation of mitochondrial aldehyde dehydrogenase. Function and regulation of monoamine enzymes: basic and clinical aspects. Proceedings of a conference held at Airlie House March 6–8 1981, London: Palgrave Macmillan UK, 1981.

[31] Koc B Kizildag S Hosgorler F Gumus H Kandis S Ates M. Magnesium citrate increases pain threshold and reduces TLR4 concentration in the brain. Biol Trace Elem Res. (2021) 199:1954–66. doi: 10.1007/s12011-020-02384-5 32989649

[32] Han S-O Kommaddi RP Shenoy SK. Distinct roles for β-arrestin2 and arrestin-domain-containing proteins in β2 adrenergic receptor trafficking. EMBO Rep. (2013) 14:164–71. doi: 10.1038/embor.2012.187 23208550 PMC3596129

[33] Cross AR Erickson RW Ellis BA. Spontaneous activation of NADPH oxidase in a cell-free system: unexpected multiple effects of magnesium ion concentrations. Biochem J. (1999) 338:229–33. doi: 10.1042/bj3380229 9931320 PMC1220046

[34] Xiao B Ma L Xiao C Lu M Xu W Huang Y. Protective effect of heat shock protein 70 and magnesium sulfate supplementation on renal ischemia reperfusion injury. Beijing Da Xue Xue Bao Yi Xue Ban. (2011) 43:525–30. PMID: 21844959

[35] Leem Y-H Lee K-S Kim J-H Seok HK Chang JS Lee DH. Magnesium ions facilitate integrin alpha 2- and alpha 3-mediated proliferation and enhance alkaline phosphatase expression and activity in hBMSCs. J Tissue Eng Regen Med. (2016) 10:E527–36. doi: 10.1002/term.1861, PMID: 24616281

[36] Wang S Zhao X Hsu Y He Y Wang F Yang F. Surface modification of titanium implants with mg-containing coatings to promote osseointegration. Acta Biomater. (2023) S1742-7061:00439–7. doi: 10.1016/j.actbio.2023.07.048 37517617

[37] Simon I Zerial M Goody RS. Kinetics of interaction of Rab5 and Rab7 with nucleotides and magnesium ions. J Biol Chem. (1996) 271:20470–8. doi: 10.1074/jbc.271.34.20470, PMID: 8702787

[38] Bao HY Wang W Sun HB Chen JZ. Binding modes of GDP, GTP and GNP to NRAS deciphered by using Gaussian accelerated molecular dynamics simulations. SAR QSAR Environ Res. (2023) 34:65–89. doi: 10.1080/1062936X.2023.2165542, PMID: 36762439

[39] Saito Y Okamoto K Kobayashi M Narumi K Yamada T Iseki K. Magnesium attenuates cisplatin-induced nephrotoxicity by regulating the expression of renal transporters. Eur J Pharmacol. (2017) 811:191–8. doi: 10.1016/j.ejphar.2017.05.034, PMID: 28529140

[40] Bellomo E Abro A Hogstrand C Maret W Domene C. Role of zinc and magnesium ions in the modulation of phosphoryl transfer in protein tyrosine phosphatase 1B. J Am Chem Soc. (2018) 140:4446–54. doi: 10.1021/jacs.8b01534, PMID: 29512390

[41] Rosenfeld SS Houdusse A Sweeney HL. Magnesium regulates ADP dissociation from myosin V. J Biol Chem. (2005) 280:6072–9. doi: 10.1074/jbc.M412717200 15579901

[42] da Silva RR da Rosa NG de Oliveira LCG Juliano MA Juliano L Rosa JC. Biochemical properties and catalytic specificity of a novel neutral serine peptidase secreted by fungus Pyrenochaetopsis sp. Appl Biochem Biotechnol. (2019) 187:1158–72. doi: 10.1007/s12010-018-2875-3, PMID: 30178205

[43] Petrović J Stanić D Bulat Z Puškaš N Labudović-Borović M Batinić B. Acth-induced model of depression resistant to tricyclic antidepressants: neuroendocrine and behavioral changes and influence of long-term magnesium administration. Horm Behav. (2018) 105:1–10. doi: 10.1016/j.yhbeh.2018.07.003, PMID: 30025718

[44] Marunaka K Furukawa C Fujii N Kimura T Furuta T Matsunaga T. The RING finger- and PDZ domain-containing protein PDZRN3 controls localization of the Mg2+ regulator claudin-16 in renal tube epithelial cells. J Biol Chem. (2017) 292:13034–44. doi: 10.1074/jbc.M117.779405, PMID: 28623232 PMC5546041

[45] Liu H Li N Jin M Miao X Zhang X Zhong W. Magnesium supplementation enhances insulin sensitivity and decreases insulin resistance in diabetic rats. Iran J Basic Med Sci. (2020) 23:990–8. doi: 10.22038/ijbms.2020.40859.9650 32952944 PMC7478262

[46] Robinson JJ. Effects of calcium and magnesium on a 41-kDa serine-dependent protease possessing collagen-cleavage activity. J Cell Biochem. (2000) 80:139–45. doi: 10.1002/1097-4644(20010101)80:1<139::AID-JCB130>3.0.CO;2-A, PMID: 11029761

[47] Chang Y-Y Lin T-Y Kao M-C Chen TY Cheng CF Wong CS. Magnesium sulfate inhibits binding of lipopolysaccharide to THP-1 cells by reducing expression of cluster of differentiation 14. Inflammopharmacology. (2019) 27:249–60. doi: 10.1007/s10787-019-00568-7, PMID: 30721372

[48] Swaminathan R. Magnesium metabolism and its disorders. Clin Biochem Rev. (2003) 24:47–66. PMID: 18568054 PMC1855626

[49] Altura BM. Basic biochemistry and physiology of magnesium: a brief review. Magnes Trace Elem. (1991) 10:167–71. PMID: 1844549

[50] Ryan MF. The role of magnesium in clinical biochemistry: an overview. Ann Clin Biochem. (1991) 28:19–26. doi: 10.1177/000456329102800103, PMID: 2024929

[51] Dominguez LJ Barbagallo M Sowers JR Resnick LM. Magnesium responsiveness to insulin and insulin-like growth factor I in erythrocytes from normotensive and hypertensive subjects. J Clin Endocrinol Metab. (1998) 83:4402–7. doi: 10.1210/jcem.83.12.5327, PMID: 9851785

[52] Guerrero-Romero F Rodríguez-Morán M. Magnesium improves the beta-cell function to compensate variation of insulin sensitivity: double-blind, randomized clinical trial. Eur J Clin Investig. (2011) 41:405–10. doi: 10.1111/j.1365-2362.2010.02422.x, PMID: 21241290

[53] Cruz KJC de Oliveira ARS Pinto DP Morais JBS da Silva Lima F Colli C. Influence of magnesium on insulin resistance in obese women. Biol Trace Elem Res. (2014) 160:305–10. doi: 10.1007/s12011-014-0044-2, PMID: 24984789

[54] Suárez A Pulido N Casla A Arrieta FJ Rovira A. Impaired tyrosine-kinase activity of muscle insulin receptors from hypomagnesaemic rats. Diabetologia. (1995) 38:1262–70. doi: 10.1007/BF00401757, PMID: 8582534

[55] Flatman PW. Mechanisms of magnesium transport. Annu Rev Physiol. (1991) 53:259–71. doi: 10.1146/annurev.ph.53.030191.001355 2042962

[56] Okahira M Kubota M Iguchi K Usui S Hirano K. Regulation of aquaporin 3 expression by magnesium ion. Eur J Pharmacol. (2008) 588:26–32. doi: 10.1016/j.ejphar.2008.03.063, PMID: 18495115

[57] Shutes A Phillips RA Corrie JET Webb MR. Role of magnesium in nucleotide exchange on the small G protein Rac investigated using novel fluorescent guanine nucleotide analogues. Biochemistry. (2002) 41:3828–35. doi: 10.1021/bi0119464 11888302

[58] Swenson AM Trivedi DV Rauscher AA Wang Y Takagi Y Palmer BM. Magnesium modulates actin binding and ADP release in myosin motors. J Biol Chem. (2014) 289:23977–91. doi: 10.1074/jbc.M114.562231, PMID: 25006251 PMC4156094

[59] Wark PA Lau R Norat T Kampman E. Magnesium intake and colorectal tumor risk: a case-control study and meta-analysis. Am J Clin Nutr. (2012) 96:622–31. doi: 10.3945/ajcn.111.030924, PMID: 22854408

[60] Lewis H Gaston K. Magnesium ions enhance the transfer of human papillomavirus E2 protein from non-specific to specific binding sites. J Mol Biol. (1999) 294:885–96. doi: 10.1006/jmbi.1999.3314, PMID: 10588894

[61] Paillet R. Chatelguyon thermal springs and amoebiasis (author’s transl). Med Trop (Mars). (1979) 39:273–7. PMID: 481180

[62] Wang J Ma X-Y Feng Y-F Ma ZS Ma TC Zhang Y. Magnesium ions promote the biological behaviour of rat Calvarial osteoblasts by activating the PI3K/Akt Signalling pathway. Biol Trace Elem Res. (2017) 179:284–93. doi: 10.1007/s12011-017-0948-8, PMID: 28205079

[63] de Sousa MSR Dos Santos LR da Cunha ST Cardoso BEP da Silva Dias TM Morais JBS. Participation of magnesium in the secretion and signaling pathways of insulin: an updated review. Biol Trace Elem Res. (2022) 200:3545–53. doi: 10.1007/s12011-021-02966-x 35666386

[64] Ehrlich B Barbagallo M Classen HG Guerrero-Romero F Mooren F Rodríguez-Morán M. Significance of magnesium in insulin resistance, metabolic syndrome, and diabetes – recommendations of the Association of Magnesium Research e.V. Trace Elements Electrolytes. (2017) 34:124–9. doi: 10.5414/TEX01473

[65] Abumaria N Yin B Zhang L Li XY Chen T Descalzi G. Effects of elevation of brain magnesium on fear conditioning, fear extinction, and synaptic plasticity in the infralimbic prefrontal cortex and lateral amygdala. J Neurosci Off J Soc Neurosci. (2011) 31:14871–81. doi: 10.1523/JNEUROSCI.3782-11.2011, PMID: 22016520 PMC6623582

[66] Xu Z-P Li L Bao J Wang ZH Zeng J Liu EJ. Magnesium protects cognitive functions and synaptic plasticity in Streptozotocin-induced sporadic Alzheimer’s model. PLoS One. (2014) 9:e108645. doi: 10.1371/journal.pone.0108645, PMID: 25268773 PMC4182554

[67] Wen J Li X. Effect of magnesium sulfate combined with Phentolamine and Nifedipine for gestational hypertension and serum levels of LIF and Apelin. J Coll Physicians Surg Pak. (2019) 29:231–4. doi: 10.29271/jcpsp.2019.03.231 30823948

[68] Tujague J Bastaki M Holland N Balmes JR Tager IB. Antioxidant intake, GSTM1 polymorphism and pulmonary function in healthy young adults. Eur Respir J. (2006) 27:282–8. doi: 10.1183/09031936.06.00033705, PMID: 16452581

[69] Saris N-EL Mervaala E Karppanen H Khawaja JA Lewenstam A. Magnesium: an update on physiological, clinical and analytical aspects. Clin Chim Acta. (2000) 294:1–26. doi: 10.1016/S0009-8981(99)00258-2 10727669

[70] Dring JC Forma A Chilimoniuk Z Dobosz M Teresiński G Buszewicz G. Essentiality of trace elements in pregnancy, fertility, and gynecologic cancers-a state-of-the-art review. Nutrients. (2021) 14:185. doi: 10.3390/nu14010185, PMID: 35011060 PMC8746721

[71] Brecht P Dring JC Yanez F Styczeń A Mertowska P Mertowski S. How do minerals, vitamins, and intestinal microbiota affect the development and progression of heart disease in adult and pediatric patients? Nutrients. (2023) 15:3264. doi: 10.3390/nu15143264, PMID: 37513682 PMC10384570

[72] Venkatesh U Sharma A Ananthan VA Subbiah P Durga R CSIR Summer Research training team. Micronutrient’s deficiency in India: a systematic review and meta-analysis. J Food Sci. (2021) 10:e110. doi: 10.1017/jns.2021.102, PMID: 35059191 PMC8727714

[73] Calder PC Carr AC Gombart AF Eggersdorfer M. Optimal nutritional status for a well-functioning immune system is an important factor to protect against viral infections. Nutrients. (2020) 12:1181. doi: 10.3390/nu12041181, PMID: 32756516 PMC7469053

[74] Han X Eggett DL Parker TL. Evaluation of the health benefits of a multivitamin, multimineral, herbal, essential oil-infused supplement: a pilot trial. J Diet Suppl. (2018) 15:153–60. doi: 10.1080/19390211.2017.1331943, PMID: 28692411

[75] Stathopoulou MG Kanoni S Papanikolaou G Antonopoulou S Nomikos T Dedoussis G. Mineral intake. Prog Mol Biol Transl Sci. (2012) 108:201–36. doi: 10.1016/B978-0-12-398397-8.00009-5 22656379

[76] McNamara AE Yin X Collins C Brennan L. Metabolomic based approach to identify biomarkers of broccoli intake. Food Funct. (2023) 14:8586–96. doi: 10.1039/d2fo03988e 37665045 PMC10508089

[77] Shim J-S Oh K Kim HC. Dietary assessment methods in epidemiologic studies. Epidemiol Health. (2014) 36:e2014009. doi: 10.4178/epih/e2014009, PMID: 25078382 PMC4154347

