# Supplementary figures and images for: Changes in the urinary proteome of rats after short-term intake of magnesium L-threonate(MgT)
Source: Front Nutr. 2023 Dec 21;10:1305738. doi: 10.3389/fnut.2023.1305738 (PMC10768015; doi:10.3389/fnut.2023.1305738)

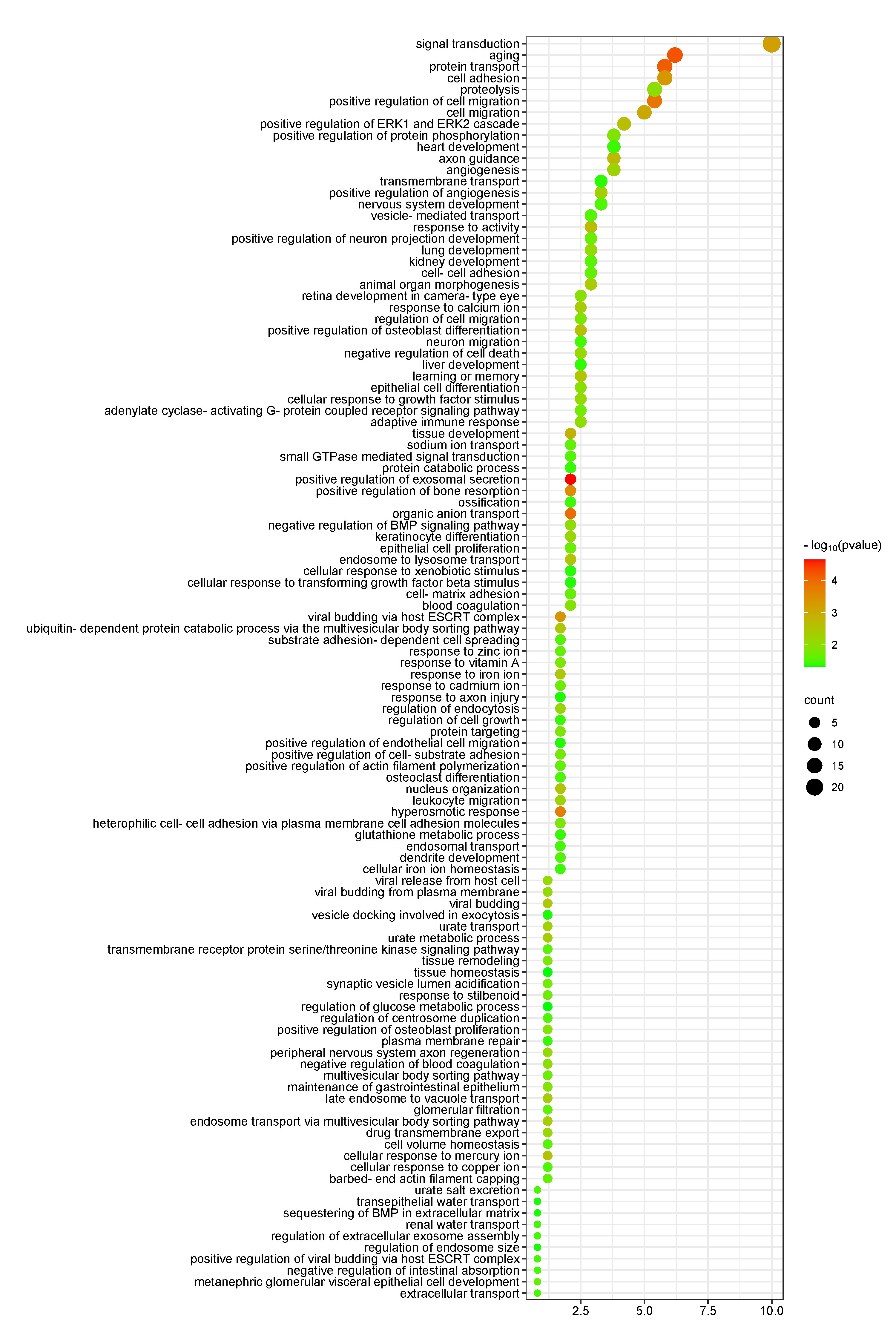

Supplement: Supplementary file 3 [file Image_2.JPEG]
